# Improvement in paediatric CT use and justification: a single-centre experience

**DOI:** 10.1093/bjro/tzae020

**Published:** 2024-08-05

**Authors:** Mariliis Tiidermann, Triin Pihlakas, Juhan Saaring, Janelle Märs, Jaanika Aasmäe, Kristiina Langemets, Mare Lintrop, Pille Kool, Pilvi Ilves

**Affiliations:** Department of Radiology, Institute of Clinical Medicine, University of Tartu, Ülikooli 18, Tartu 50090, Estonia; Radiology Clinic, Tartu University Hospital , L. Puusepa 8, Tartu 50406, Estonia; Department of Radiology, Institute of Clinical Medicine, University of Tartu, Ülikooli 18, Tartu 50090, Estonia; Radiology Clinic, Tartu University Hospital , L. Puusepa 8, Tartu 50406, Estonia; Radiology Clinic, Tartu University Hospital , L. Puusepa 8, Tartu 50406, Estonia; Radiology Clinic, Tartu University Hospital , L. Puusepa 8, Tartu 50406, Estonia; Radiology Clinic, Tartu University Hospital , L. Puusepa 8, Tartu 50406, Estonia; Radiology Clinic, Tartu University Hospital , L. Puusepa 8, Tartu 50406, Estonia; Department of Radiology, Institute of Clinical Medicine, University of Tartu, Ülikooli 18, Tartu 50090, Estonia; Radiology Clinic, Tartu University Hospital , L. Puusepa 8, Tartu 50406, Estonia; Department of Radiology, Institute of Clinical Medicine, University of Tartu, Ülikooli 18, Tartu 50090, Estonia; Department of Radiology, Institute of Clinical Medicine, University of Tartu, Ülikooli 18, Tartu 50090, Estonia; Radiology Clinic, Tartu University Hospital , L. Puusepa 8, Tartu 50406, Estonia

**Keywords:** justification, appropriateness, referral, guidelines, paediatric, computed tomography, CT, magnetic resonance imaging, MRI, radiation protection

## Abstract

**Objectives:**

To analyse changes in the use of paediatric (≤16 years) CT over the past decade and to evaluate the appropriateness of CT examinations at a tertiary teaching hospital.

**Methods:**

Data from 290 paediatric CTs were prospectively collected in 2022 and compared with data from 2017 (358 cases) and 2012 (538 cases). The justification of CTs was evaluated with regard to medical imaging referral guidelines and appropriateness rates were calculated.

**Results:**

Paediatric CTs decreased 39.4% over the 10 years, contrasting with a 27.6% increase in overall CTs. Paediatric CTs as the share of overall CTs dropped from 2.5% in 2012 to 1.1% in 2022 (*P* < .0001), with a concurrent rise in paediatric MRIs (*P* < .0001). Notable reductions in CT use occurred for head trauma (*P* = .0003), chronic headache (*P* < .0001), epilepsy (*P* = .037), hydrocephalus (*P* = .0078), chest tumour (*P* = .0005), and whole-body tumour (*P* = .0041). The overall appropriateness of CTs improved from 73.1% in 2017 to 79.0% in 2022 (*P* = .0049). In 15.4% of the cases, no radiological examination was deemed necessary, and in 8.7% of the cases, another modality was more appropriate. Appropriateness rates were the highest for the head and neck angiography (100%) and the chest (96%) and the lowest for the neck (66%) and the head (67%).

**Conclusions:**

Justification of CT scans can be improved by regular educational interventions, increasing MRI accessibility, and evaluating the appropriateness of the requested CT before the examination. Interventions for a more effective implementation of referral guidelines are needed.

**Advances in knowledge:**

The focus for improvement should be CTs for head and cervical spine trauma, accounting for the majority of inappropriate requests in the paediatric population.

## Introduction

Over the last decades, the number of CT examinations has increased worldwide.^1^ It has raised concerns about higher patient exposure to ionizing radiation, particularly in the paediatric population, because children are at a higher risk for long-term stochastic effects for tumours.^2–6^

According to the International Safety Standards set by the International Atomic Energy Agency (IAEA), CT examinations involving exposure to ionizing radiation must be both justified and optimized.^5^ There is growing emphasis on implementing the principle of justification for medical exposures in Europe, as advocated by the European Union (EU),^4^ national radiological protection competent authorities (eg, HERCA^7^^,^^8^), and professional societies.^9^

A CT study is considered ‘justified’ when the expected benefits outweigh the radiation-related risks.^10^ From the standpoint of radiation protection, the best radiation protection is to avoid a CT examination whenever possible.^11^ Alternative modalities that can answer the clinical question with lower dose or no radiation should be considered. Evidence-based referral guidelines for medical imaging aid in deciding whether a planned CT in a particular clinical situation is indicated. Appropriate imaging referrals not only reduce population radiation exposure but, importantly, also conserve valuable healthcare resources.^12^

## Objectives

The objectives of this study were to analyse changes in the use of paediatric CT over the past 10 years and to evaluate the appropriateness of CT examinations in accordance with the referral guidelines for medical imaging at a tertiary teaching hospital.

## Methods

### Data collection

In 2022, data about all CT examinations performed in children aged 16 years and younger were prospectively collected at an academic tertiary regional hospital, which serves as one of the two third-level children’s hospitals in the country. Data from similar studies conducted at the same hospital in the years 2017 and 2012 were used for comparison.^13^

Patients’ age, gender and weight, clinical information from CT request, the clinical question to be answered, the body region(s) scanned, the imaging protocol selected, the use of contrast agent, and the number of phases were recorded in a survey questionnaire. The final diagnosis was collected from the radiology report. The total number of CT and MRI scans were obtained from the nationwide picture archiving and communication system.

### Appropriateness of CT examinations

All CT examinations from the years 2017 and 2022 were evaluated for the justification of radiation doses. Retrospective evaluation for the year 2012 was not possible due to the lack of data on the appropriateness of CTs, limited data availability, and different appropriateness criteria and imaging referral guidelines in place back then.

Since there are no national referral guidelines for medical imaging, the hospital makes use of the international clinical decision support system, UpToDate.^14^ The appropriateness of the selected anatomical region(s) and phase(s) for each CT request was therefore evaluated based on the referral guidelines provided by UpToDate.

Clinical details justifying the performance and appropriate selection of the examination were collected from the referral. When available information was insufficient, additional clinical details were drawn from the electronic health record. The selected anatomical region or phase was considered indicated when (1) criteria for doing a CT scan were fulfilled with regard to the referral guideline based on clinical information, and (2) it answered the clinical question with the lowest achievable radiation exposure.

In cases where no recommendation was found in the referral guidelines for a specific clinical case, researchers relied on their own professional judgement. If a scan was deemed inappropriate, additional questions were addressed, including whether more clinical information for justification was necessary, whether another examination would have been more appropriate, and if so, which type of examination would have been more appropriate. Two researchers independently evaluated the same sample of requests. In cases of discrepancy, a consensus search was conducted. If persistent disagreement occurred between the 2 researchers, the request was excluded from data analysis.

The appropriateness rate (AR) was defined as the ratio of appropriate requests to the total number of analysed requests in the group.

### Data analysis

Statistical analysis was conducted using the statistical package SAS version 9.4 (Copyright (c) 2002-2012 by SAS Institute Inc., Cary, NC, USA) and RStudio. Statistical comparison between ARs in the years 2017 and 2022 was made using the chi-square test. Odds ratios (OR) and 95% CI were calculated to estimate differences.

## Results

**Figure 1.**
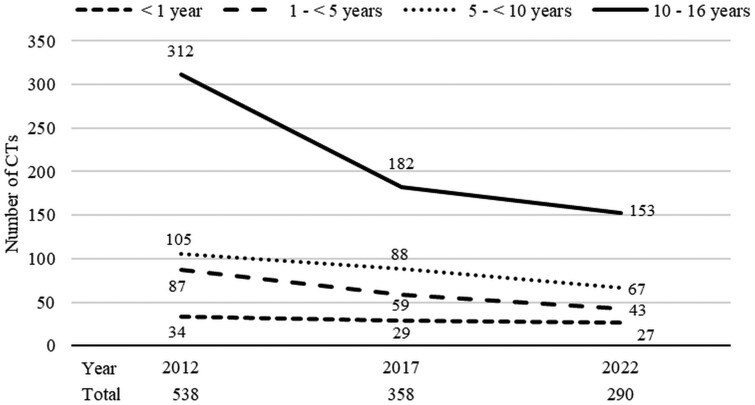
Number of the paediatric CT examinations in each age group and in total for 2012, 2017, and 2022.

In 2022, data about 290 paediatric CT examinations were collected. Including data from previous studies (358 in 2017 and 538 in 2012), a total of 1186 paediatric CT examinations were analysed (Figure 1). Among these, 59.8% (*n* = 709) were performed in male patients. The age distribution was the following: 54% (*n* = 647) in the group of 10-16 years, 22% (*n* = 260) in the group of 5 to <10 years, 16% (*n* = 189) in the group of 1 to <5 years, and 7% (*n* = 90) in the group of <1 year. No statistically significant differences were found in age distribution when comparing the years 2012, 2017, and 2022 (overall *P* = .26).

**Figure 2.**
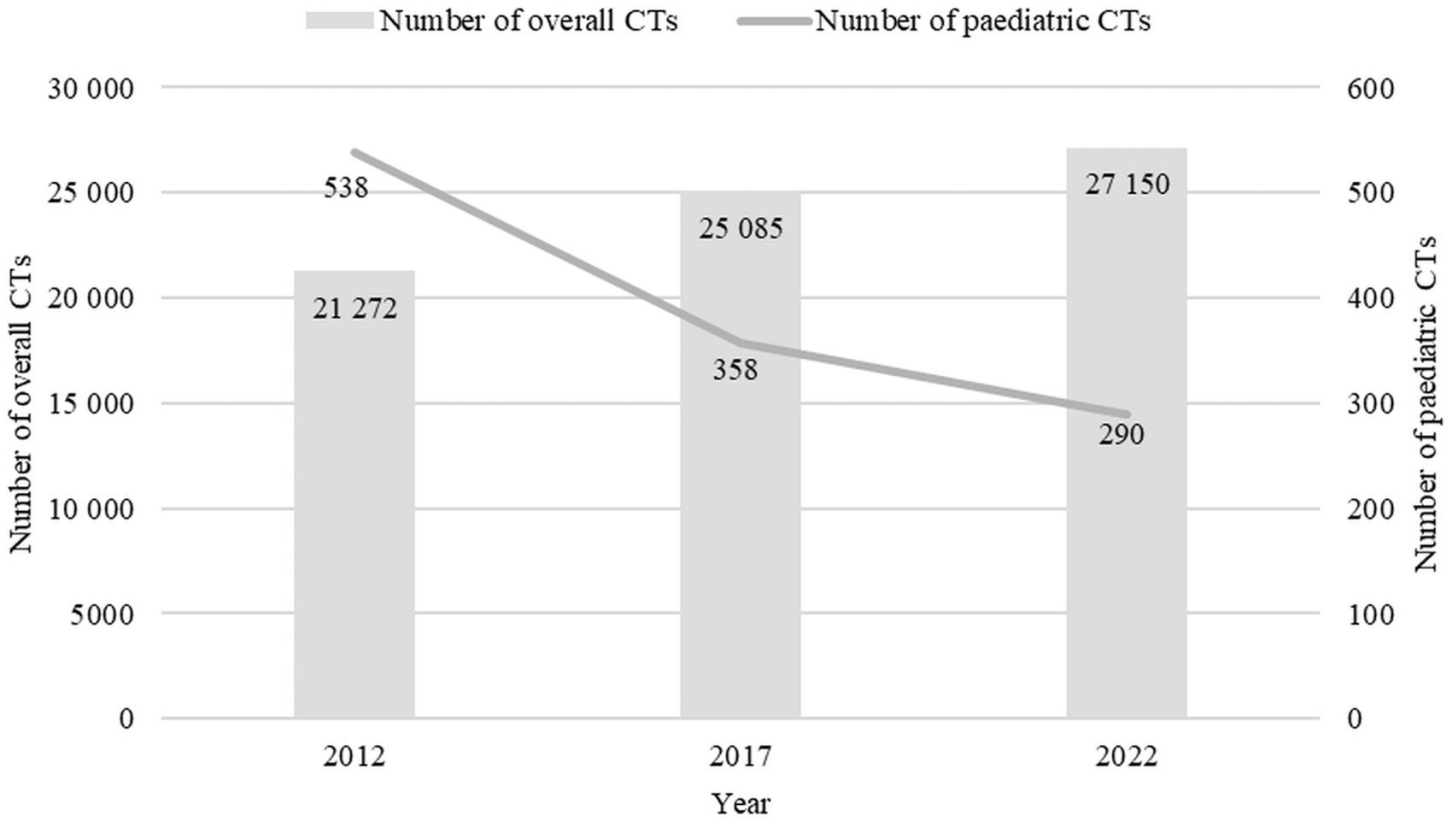
Number of the overall and paediatric CTs for 2012, 2017, and 2022.

Over the past 10 years, the number of paediatric CTs decreased 1.7 times (39.4%), while the overall number of CTs increased 1.3 times (27.6%) (Figure 2). Consequently, the share of paediatric CTs in overall CTs decreased from 2.5% (538 paediatric CTs out of 21 272 overall CTs) in 2012 to 1.1% (290 paediatric CTs out of 27 150 overall CTs) in 2022 (*P* < .0001; OR = 2.40, 95% CI, 2.08-2.79).

**Figure 3.**
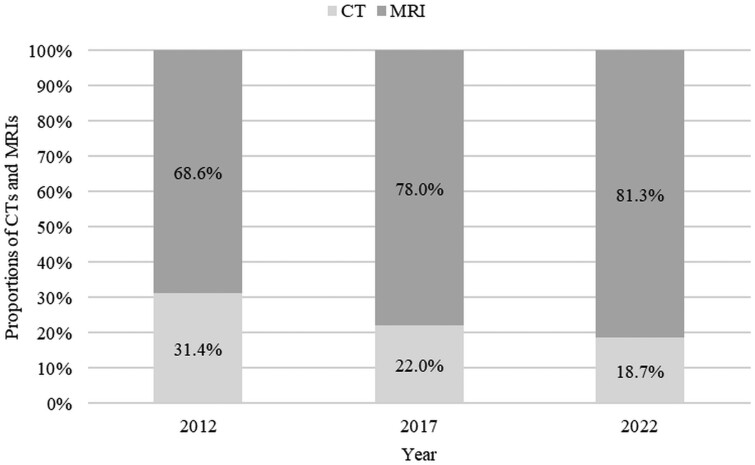
Proportions of the CT and MRI examinations in the paediatric population for 2012, 2017, and 2022. Overall *P* < .0001.

At the same time, the number of paediatric MRIs increased from 1175 in 2012 to 1260 in 2022 (7.2%). Consequently, the study revealed a significant change in the proportions of paediatric CT and MRI scans; it demonstrated a significant increase in the use of MRIs and a decrease in CTs over 10 years (overall *P* < .0001) (Figure 3). In 2012, almost one-third (31.4%) of all CT and MRI examinations in children were CTs (538 CTs versus 1175 MRIs, total 1713). By 2017, the proportion of CTs had decreased to 22.0% (358 CTs versus 1266 MRIs, total 1624), and by 2022, only 1 in every 5 (18.7%) examinations was a CT (290 CTs versus 1260 MRIs, total 1550).

The summary of the anatomical regions scanned and the clinical indications used is presented in Tables 1 and 2. A total of 1475 distinct anatomical CT examinations were received. The head was the most frequently scanned anatomical region (776/1475; 52.6%), primarily due to trauma (404/1475; 27.4%), followed by routine (all indications excluding trauma) examinations of the head (372/1475; 25.2%) (Table 1). CT for cervical spine trauma was conducted in 8.3% (123/1475) of the cases, and CT for the chest anomaly accounted for 4.8% (66/1475) of the cases (Table 2).

**Table 1.** Number of the head CT examinations grouped by the clinical indications used for 2012, 2017, 2022 and in total.

	Trauma	Tumour	Infection	Congenital anomaly	Chronic headache	Epilepsy	Hydrocephalus	Stroke	Other	Total
2012	178	31	15	18	84	21	19	2	22	393
2017	115^a^	11	20^a^	5	7^a^	6	2^a^	32^a^	7	205^a^
2022	111^b^	7	11	10	1^b^	2^b^	2^b^	30^b^	4	178^b^
Total	404	49	46	33	92	29	23	67	33	776
Overall *P* value	.0003	.16	.014	.28	<.0001	.037	.0078	<.0001	.15	<.0001

a *P* ≤ .05 2012 vs 2017.

b *P* ≤ .05 2012 vs 2022.

c *P* ≤ .05 2017 vs 2022.

**Table 2.** Number of the body CT examinations grouped by the clinical indications used for 2012, 2017, 2022 and in total.

	Trauma	Tumour	Infection	Congenital anomaly	Other
**Neck**					
2012	43	2	2	4	5
2017	39	1	1	5^a^	0
2022	41	0	7	0	0
Total	123	3	10	9	5
Overall *P* value	.44	.64	.047	.047	.012
**Chest**					
2012	4	14	16	9	6
2017	4	5^a^	14	26^a^	17
2022	2	3^b^	13	31^b^	7
Total	10	22	43	66	30
Overall *P* value	.60	.0005	.35	.0005	.082
**Abdomen-pelvis**					
2012	6	6	9	1	0
2017	9	2	7	0	1
2022	2^c^	2	9	3	1
Total	17	10	25	4	2
Overall *P* value	.062	.38	.60	.08	.52
**Whole body (chest, abdomen-pelvis)**					
2012	8	10	1	0	0
2017	15^a^	5	0	0	0
2022	21	3^b^	3	0	3
Total	44	18	4	0	3
Overall *P* value	.065	.0041	.38		.25
**Thoracic or lumbar spine**					
2012	12	0	0	0	1
2017	8	0	0	0	2
2022	5	0	0	0	0
Total	25	0	0	0	3
Overall *P* value	.56				.56
**Pelvic bones**					
2012	3	0	0	4	0
2017	0	0	0	1	0
2022	2	0	0	1	0
Total	5	0	0	6	0
Overall *P* value	>.99			>.99	
**Extremity**					
2012	14	0	1	0	2
2017	16	1	0	6^a^	0
2022	2	1	0	0	0
Total	32	2	1	6	2
Overall *P* value	.56	.14	.47	.068	.29

a *P* ≤ .05 2012 vs 2017.

b *P* ≤ .05 2012 vs 2022.

c *P* ≤ .05 2017 vs 2022.

In the first 5 years, the number of CTs for head trauma decreased by 35.4%, from 178 in 2012 to 115 in 2017, and remained relatively stable thereafter (115 head trauma CT scans in 2017 and 111 in 2022). In 2012, performing head CT due to chronic headache, epilepsy, or hydrocephalus was common, but these indications significantly decreased by 2017 and 2022 (overall *P* < .0001 for headache; overall *P* = .037 for epilepsy; overall *P* = .0078 for hydrocephalus) (Table 1).

Also the number of CT requests for chest and whole-body (eg, chest, abdomen, and pelvis) tumours decreased when comparing the years 2012, 2017, and 2022 (overall *P* = .0005 for chest tumours; overall *P* = .0041 for whole-body tumours) (Table 2). At the same time, the number of cervical spine CT requests for trauma remained relatively stable during the study period (overall *P* = .44). The frequency of head CT for stroke in an acute setting showed an increase (overall *P* < .0001) (Table 1).

The number of phases used is presented in Table 3. Single-phase CT scan was performed in 93.9% (1303/1388) of the cases, representing a favourable outcome in terms of radiation protection (ie, minimizing radiation exposure). There was no significant change in the rate of single or multiple-phase investigations over the past 10 years (overall *P* = .59). In 2012, the investigation of brain tumour was the most frequent two-phase examination, accounting for 22% (8/36) of these examinations. In 2017 and 2022, the investigation of stroke (head CT without contrast and CT angiography of the head arteries or the head and neck arteries) accounted for 31% (9/29) and 55% (11/20) of two-phase examinations, respectively. Three-phase examinations were very rare: only one abdominal CT for trauma in 2017 and one chest CT for congenital anomaly in 2022. No four-phase examinations were conducted.

**Table 3.** Number of the single-phase and multiphase CT examinations for 2012, 2017, 2022 and in total.

	One phase	Two phases	Three phases	Overall *P* value
2012	575/611 (94.1%)	36/611 (5.9%)	0	
2017	388/417 (93.0%)	28/417 (6.7%)	1/417 (0.2%)	
2022	340/360 (94.4%)	19/360 (5.3%)	1/360 (0.3%)	
Total	1303/1388 (93.9%)	83/1388 (6.0%)	2/1388 (0.1%)	.59

### Appropriateness of CT examinations

**Figure 4.**
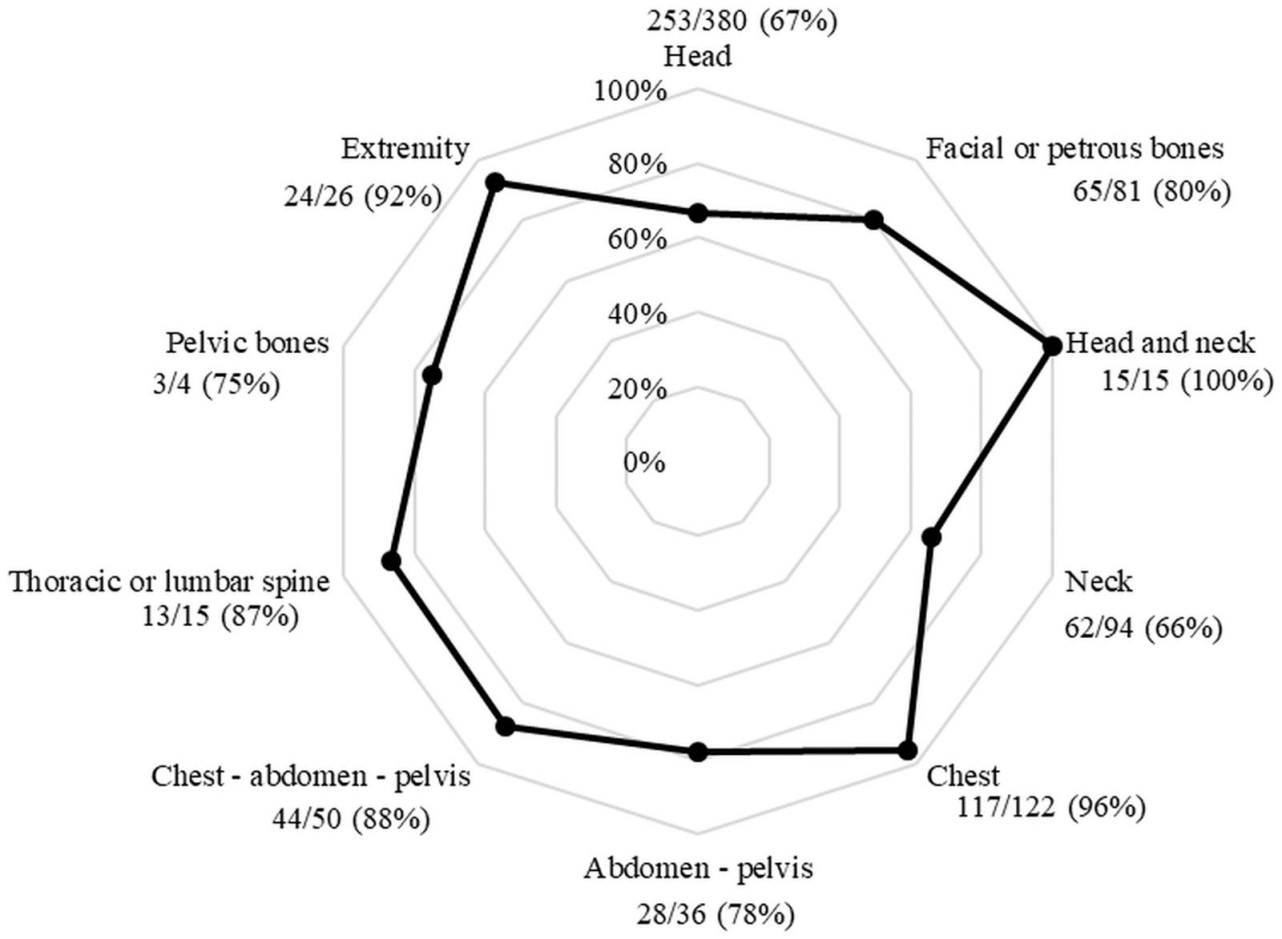
Total appropriateness rates of the CT scans according to the anatomical areas for 2017 and 2022 combined. Appropriateness rate—the ratio of the number of appropriate scans in the group to the total number of scans in the group.

The clinical elements for justification were not present, or consensus was not found in 4 cases, which were subsequently excluded from further analysis. The total analysed sample consisted of 824 scans. The requested examinations were considered appropriate in 75.8% (625/824) of the cases (Table 4). Comparison of ARs between 2017 and 2022 revealed improvement from 73.1% (324/443) in 2017 to 79.0% (301/381) in 2022 (*P* = .0499; OR = 1.4, 95% CI, 1.00-1.91). Total ARs of the CT scans according to the anatomical areas are shown in Figure 4. The AR was the lowest for the neck (62/94; 66%), followed by the head (253/380; 67%). The highest AR was observed for investigations of the chest (117/122; 96%), and the head and neck angiography (15/15; 100%).

**Table 4.** Appropriateness of the CT scans for 2017, 2022 and in total.

	2017	2022	Total	2017 vs 2022	
	*n*/*N* (AR%)	*n*/*N* (AR%)	*n*/*N* (AR%)	*P* value	OR (95% CI)
Appropriate	324/443 (73.1)	301/381 (79.0)	625/824 (75.8)	.0499	1.4 (1.00-1.91)
Inappropriate	119/443 (26.9)	80/381 (21.0)	199/824 (24.1)		
No examination proposed	71/119 (59.7)	56/80 (70.0)	127/199 (63.8)	.14	
Another modality more appropriate	48/119 (40.3)	24/80 (30.0)	72/199 (36.2)	.14	
MRI	36/48 (75.0)	17/24 (70.8)	53/72 (73.6)	.71	
Ultrasound	2/48 (4.2)	3/24 (12.5)	5/72 (6.9)	.33	
Radiography	7/48 (14.6)	4/24 (16.7)	11/72 (15.3)	>.99	
PET	3/48 (6.3)	0/24 (0.0)	3/72 (4.2)	.55	

Abbreviations: AR = appropriateness rate (%)—the ratio of the number of appropriate scans in the group to the total number of analysed scans in the group; *n* = number of appropriate scans; *N* = number of all scans.

Appropriateness according to the anatomical areas and clinical indications is presented in Table 5. The AR was excellent (100%) in the case of CT requests for head CT for anomaly, paranasal CT for chronic sinusitis, CT angiography of the head and neck arteries for stroke, and cardiovascular CT angiography. The AR was very low for chronic headache (0%), brain tumour (11%), and hydrocephalus (25%); low for head trauma (61%), epilepsy (63%), and cervical spine trauma (64%). The most significant improvement in AR between 2017 and 2022 was noted for whole-body CTs which increased from 70% to 100% (*P* = .0024), and for head routine (all indications excluding trauma) CTs which improved from 67% in 2017 to 82% in 2022 (*P* = .031). In all other categories, justification remained nearly constant.

**Table 5.** Appropriateness of the CT scans according to the anatomical area and clinical indication for 2017, 2022 and in total.

	2017	2022	Total	2017 vs 2022	
	*n*/*N* (AR%)	*n*/*N* (AR%)	*n*/*N* (AR%)	*P* value	OR (95% CI)
Head: trauma	66/113 (58.4)	72/111 (64.9)	138/224 (61.6)	.32	
Head: routine	60/89 (67.4)	55/67 (82.1)	115/156 (73.7)	.039	2.2 (1.03-4.8)
Tumour	1/11 (9.1)	1/7 (14.3)	2/18 (11.1)	>.99	
Infection	17/20 (85.0)	11/11 (100.0)	28/31 (90.3)	.54	
Congenital anomaly	5/5 (100.0)	10/10 (100.0)	15/15 (100.0)	NA	
Chronic headache	0/7 (0.0)	0/1 (0.0)	0/8 (0.0)	NA	
Epilepsy	3/6 (50.0)	2/2 (100.0)	5/8 (62.5)	.46	
Hydrocephalus	0/2 (0.0)	1/2 (50.0)	1/4 (25.0)	>.99	
Stroke	29/32 (90.6)	28/30 (93.3)	57/62 (91.9)	>.99	
Other	5/6 (83.3)	2/4 (50.0)	7/10 (70.0)	.50	
Facial bones: trauma	7/8 (87.5)	14/17 (82.4)	21/25 (84.0)	>.99	
Paranasal sinuses: chronic sinusitis	14/14 (100.0)	6/6 (100.0)	20/20 (100.0)	NA	
Petrous bone	10/17 (58.8)	3/8 (37.5)	13/25 (52.0)	.41	
Cervical spine: trauma	22/39 (56.4)	29/41 (70.7)	51/80 (63.8)	.18	
Neck soft tissue	6/7 (85.7)	5/7 (71.4)	11/14 (78.6)	>.99	
Head and neck CTA: stroke	7/7 (100.0)	8/8 (100.0)	15/15 (100.0)	NA	
Chest (excluding cardiovascular)	55/59 (93.2)	48/49 (98.0)	103/108 (95.4)	0.37	
Chest: cardiovascular CTA	7/7 (100.0)	7/7 (100.0)	14/14 (100.0)	NA	
Abdomen-pelvis	15/19 (78.9)	13/17 (76.5)	28/36 (77.8)	>.99	
Chest, abdomen-pelvis	14/20 (70.0)	30/30 (100.0)	44/50 (88.0)	.0024	27 (1.4-519)
Thoracic or lumbar spine	9/10 (90.0)	4/5 (80.0)	13/15 (86.7)	>.99	
Pelvic bones	1/1 (100.0)	2/3 (66.7)	3/4 (75.0)	>0.99	
Extremity	21/23 (91.3)	3/3 (100.0)	24/26 (92.3)	>.99	
Total	324/443 (73.1)	301/381 (79.0)	625/824 (75.8)	.0499	1.4 (1.00-1.91)

Head routine includes following clinical indications: tumour, infection, congenital anomaly, chronic headache, epilepsy, hydrocephalus, stroke, and other.

Abbreviations: AR = appropriateness rate (%)—the ratio of the number of appropriate scans in the group to the total number of analysed scans in the group; *n* = number of appropriate scans; *N* = number of all scans.

In cases of inappropriate requests (199/824; 24.1%), no radiological examination was deemed necessary in 127/199 (64%) of the cases (Table 4). Two-thirds (86/127; 68%) of these cases were head trauma, followed by cervical spine trauma (29/127; 23%). None of the patients with an inappropriate request had any traumatic intracranial findings (head CT) or cervical spine trauma (cervical spine CT). Another modality with a lower dose or a non-ionizing modality would have been more appropriate in 72/199 (36%) inappropriate requests; of these, MRI in 74% (53/72), radiography in 15% (11/72), sonography in 7% (5/72), and PET in 4% (3/72) of the cases.

Native or another phase with contrast media was unjustified in 6/49 (12%) multiphase examinations.

## Discussion

Our study demonstrates that, despite the overall increase in the number of CT examinations at the academic tertiary regional hospital, serving as one of the two third-level children’s hospitals in the country, the number of paediatric CT scans has significantly decreased over the past 10 years. The proportion of paediatric CT scans in all paediatric CT and MRI examinations has also significantly decreased. There has been a notable reduction in the number of CTs for head trauma, chronic headache, epilepsy, hydrocephalus, chest tumour, and whole-body tumour. While the overall appropriateness of paediatric CT examinations is not satisfactory, it has shown improvement increasing from 73.1% in 2017 to 79.0% in 2022.

This positive trend among the paediatric population can be attributed to several reasons. Since 2012, considerable effort has been put into raising the awareness of the ALARA (‘As Low As Reasonably Achievable’) principle, as well as the potential long-term risks of radiation exposure in the hospital. Regular educational and awareness lectures have been conducted not only in the radiology department for radiographers and radiologists but also in various referring departments, for medical students and residents, and at national meetings of various organizations. Consultation between referring physician and radiologist was made obligatory before performing a paediatric CT scan to ensure the advance justification of CT. As a result, healthcare providers have become more cautious about using CT scans for paediatric patients.

Additionally, diagnostic capabilities have improved and alternative imaging modalities are being used more frequently. Compared to 2012, a significant decrease in the number of CT studies and a notable increase in MRI examinations had occurred by 2017, while the number of MRI units remained the same, 2. This suggests improvement in the accessibility of MRI examinations even with limited resources and the increased awareness of the ALARA principle. By the year 2022, the number of MRI units had doubled from 2 to 4. At present, children with headache, epilepsy, and hydrocephalus are almost always investigated with MRI.

Since 2018, with the implementation of cone-beam CT (CBCT) for skeletal investigations at the hospital, almost all extremity examinations have been conducted using CBCT, which involves a lower radiation dose compared to CT. In 2022, only 3 extremity CTs were performed: 2 trauma shoulder CTs, as the shoulder cannot be examined by using CBCT due to technical reasons, and 1 femoral CT for planning microwave ablation of osteoid osteoma.

Although there was an increase in the overall appropriateness of CT requests from 2017 to 2022, their justification is still suboptimal. In 2022, 21% of all paediatric CT examinations were inappropriate, which is of concern regarding the unjustified exposure of paediatric patients to ionizing radiation. Similar findings have been reported in previous studies. While the ARs of paediatric CT examinations are higher than among the adult population, they are still lower than ideal, with inappropriate examinations ranging from 13% to 35%.^15–19^ A recent appropriateness study from Sweden^19^ did not show improvement in CT requests over the last 15 years in the adult population. In contrast, in Finland,^16^^,^^17^ it was found that the proportion of justified CT examinations in children and young adults up to 35 years of age increased from 71% to 87% during 4 years through regular education, increased MRI capacity, and the implementation of imaging referral guidelines.

According to a recent EU-initiated CT justification project,^12^ imaging referral guidelines, whether local, national, or adapted from another country, are widely available (63%) in Europe. However, only 13% of healthcare professionals consider them in daily use. The European Society of Radiology—EuroSafe Imaging survey^20^ revealed a limited availability of dedicated guidelines for children and pregnant women (39% in EU/EEA and 30% in non-EU-EEA countries). This confirms the lack of effective and widespread adoption of imaging referral guidelines, particularly among children.^12^

It appeared the guidelines provided recommendations for almost all clinical cases observed in our study. The lowest rate of justified CTs was observed for cases of head and neck trauma (67% and 66%, respectively) and there occurred no statistically significant improvement. This indicates that the guidelines are not adequately used or followed at the studied hospital. Multidisciplinary cooperation and more effective implementation of referral guidelines in daily practice would aid in improving the appropriateness of these investigations.

Furthermore, our study demonstrates the reliability of paediatric referral guidelines for medical imaging. For example, the negative predictive value for head and neck trauma was 100%, consistent with the findings of other studies^21^ and was also confirmed in our study. This indicates that medical imaging referral guidelines are appropriate for guiding in justification of CT examinations.

The reasons for the limited use of referral guidelines can be complex, as has been highlighted in the reports of the IAEA^22^ and the recent EU-initiated CT justification project.^12^ These reasons may include limited access or familiarity with internationally recognized referral guidelines for medical imaging, difficulties in integrating referral guidelines into the clinical decision support system interfacing with electronic requesting systems, lack of national referral guidelines, frequent use of radiology for defensive medicine, communication failures among healthcare professionals, work pressure, and inertia among healthcare professionals who may feel that not much can be done to improve the situation. These factors combined can play a major role and explain the poor implementation of paediatric guidelines at the studied hospital.

The major limitation of this study is that it relies on data from a single institution, and the number of paediatric CTs is relatively low. A multicentre or nationwide study would provide more comprehensive information. Nevertheless, the study encompasses paediatric CT cases at one of the two tertiary children's hospitals of the country over a 10-year period, evaluating the changes that took place during that time. However, data on the appropriateness of CTs from the year 2012 was lacking and therefore excluded from the analyses of changes in justification.

One of the challenges to be faced was the assessment of justification of CTs for various anatomical regions and indications in a situation where national guidelines were not available. Only 2 researchers defined the appropriateness of the CT exams; however, the inter-researcher agreement was high.

## Conclusion

Over the past 10 years, the number of paediatric CT scans has significantly decreased despite the overall rise in the number of CTs, and the number of paediatric MRIs has increased. The total appropriateness of paediatric CT examinations is not satisfactory, but it has improved reaching 79%. Collective efforts to further develop the justification of paediatric CTs should continue. Regular educational and awareness interventions, increasing the capacity and accessibility of MRI examinations, and using advanced justification help improve the appropriateness of CT examinations. The use of referral guidelines in daily practice is limited and challenging, and the reasons for this can be complex. Multidisciplinary cooperation and interventions are needed for a more effective implementation of referral guidelines. The focus for improvement should be CT examinations for head and cervical spine trauma, as these account for the majority of inappropriate requests in the paediatric population.

## Supplementary Material

tzae020_Supplementary_Data
